# A Survey of Online Activity Recognition Using Mobile Phones

**DOI:** 10.3390/s150102059

**Published:** 2015-01-19

**Authors:** Muhammad Shoaib, Stephan Bosch, Ozlem Durmaz Incel, Hans Scholten, Paul J.M. Havinga

**Affiliations:** 1 Pervasive Systems Group, Department of Computer Science, Zilverling Building, PO-Box 217, 7500 AE Enschede, The Netherlands; E-Mails: stephan@inertia-technology.com (S.B.); hans.scholten@utwente.nl (H.S.); p.j.m.havinga@utwente.nl (P.J.M.H.); 2 Department of Computer Engineering, Galatasaray University, Ortakoy, Istanbul 34349, Turkey; E-Mail: odincel@gsu.edu.tr

**Keywords:** online activity recognition, real time, smartphones, mobile phone, mobile phone sensing, human activity recognition review, survey, accelerometer

## Abstract

Physical activity recognition using embedded sensors has enabled many context-aware applications in different areas, such as healthcare. Initially, one or more dedicated wearable sensors were used for such applications. However, recently, many researchers started using mobile phones for this purpose, since these ubiquitous devices are equipped with various sensors, ranging from accelerometers to magnetic field sensors. In most of the current studies, sensor data collected for activity recognition are analyzed offline using machine learning tools. However, there is now a trend towards implementing activity recognition systems on these devices in an online manner, since modern mobile phones have become more powerful in terms of available resources, such as CPU, memory and battery. The research on offline activity recognition has been reviewed in several earlier studies in detail. However, work done on online activity recognition is still in its infancy and is yet to be reviewed. In this paper, we review the studies done so far that implement activity recognition systems on mobile phones and use only their on-board sensors. We discuss various aspects of these studies. Moreover, we discuss their limitations and present various recommendations for future research.

## 1. Introduction

Human activity recognition has enabled novel applications in different areas, such as, healthcare, security and entertainment [1,2]. Initially, dedicated wearable motion sensors were used to recognize different physical activities [1–5]. However, there has been a shift towards mobile phones in recent years, because of the availability of various sensors in these devices. Examples of such sensors are GPS, accelerometer, gyroscope, microphone and magnetometer.

Most of the research on human activity recognition using mobile phones is done offline in machine learning tools, such as WEKA [6–11]. Mobile phones were initially considered as resource-limited devices [12]. For example, they did not possess enough battery resources (lower mAh) to run activity recognition systems for an extended period. Moreover, it is a challenging task to implement and evaluate different recognition systems on these devices. However, in recent years, mobile phones have become capable of running such recognition systems, so there has been a shift towards online activity recognition. For example, we have shown in Section 3.4 that for various mobile phones, the battery capacities have increased from 950 mAh in 2008 to 1500 mAh in 2013. There are a number of studies where activity recognition has been implemented on mobile phones for real-time processing. In some of these studies, the aim is to show that online recognizers can work on mobile phones considering the available resources, while in other studies, the aim is to develop an application where the activities of the users can be tracked, such as a mobile diary or a fitness tracker [13].

There is a number of survey publications that have reviewed the work done so far in this area [1,14–17]. Though these surveys have partially covered online activity recognition, their focus is mainly on studies with offline analysis. Moreover, they are generic studies covering different aspects of context-aware applications on mobile phones and wearable sensors. To the best of our knowledge, there is no survey that focuses only on the online activity recognition using solely the mobile phone sensors. By online activity recognition, we mean that the data collection, preprocessing and classification steps are done locally on the mobile phone. In some cases, the online activity recognition is done on a remote server or in a cloud, we do not consider such studies, as discussed in Section 3. Because we report all studies done so far on online activity recognition using mobile phones only, we believe that this study will help researchers in the future work in this area of research. It is important to note that “online activity recognition on smartphones” should not be confused with “online machine learning models”. “Online machine learning models” are able to adapt themselves according to new data points, unlike offline or batch learning models [18]. The details of online *vs.* offline learning models can be found in [18], but this is not the focus of our paper. We use the “online” term in a different way, for the practical implementation of activity recognition systems on mobile phones. These implemented systems can be using either an online or a batch learning model.

In this paper, we focus on the work in which such systems have been implemented on mobile phones. We are interested in systems that can recognize different physical activities. For comparing these studies, we use different criteria, such as classification methods, experimental setups, position and orientation independence, real-time feedback, assistive feedback, evaluation methods, dynamic and adaptive sensor selection, adaptive sampling and resource consumption analysis. Because we are only interested in studies that focus on online activity recognition using mobile phones, we used the following criteria for the selection of the studies reviewed in this paper:

- They implement the activity recognition system fully on mobile phones, such that sensing, preprocessing and classification are all done locally on these devices.
- They use only mobile phone sensors, where motion sensors are used as the main sensors in the recognition process. For example, we did not include [19] in our review, because it uses an external motion sensor for physical activity recognition in combination with a mobile phone accelerometer. However, we consider studies that use other on-board sensors as additional sensors, such as a microphone, gyroscope, GPS, and pressure sensor.
- They are able to recognize different physical activities. We do not include studies on fall detection and posture detection in this work.

There are also some studies [19–25] that have reported that they have implemented online activity recognition, but we could not find evaluation proof or details for such a claim in the respective papers. Therefore, we did not include these studies in our review.

The rest of the paper is organized as follows. In Section 2, we briefly describe the related work. A comparison of all reported studies on online activity recognition is described in Section 3. In Section 4, we discuss possible improvements to the current research and future directions. Finally, we conclude this paper in Section 5.

## 2. Related Work

Human activity recognition using wearable sensors is a very broad research subject. Earlier work by Lara *et al.* [1], Akker *et al.* [16] and Preece *et al.* [26] provides an outline of relevant research and applicable techniques. These surveys include all wearable solutions. In contrast, our survey focuses on human activity recognition solutions using a specific wearable sensor platform: the smart phone. The smart phone is quickly gaining popularity as a wearable sensor platform. It is being applied for many applications, including health monitoring, monitoring road and traffic conditions, commerce, environmental monitoring and recognizing human behavior [27]. Earlier work by Incel *et al.* [14] surveys activity recognition research using smart phones. However, most research described therein still involves offline processing of the data collected on the smart phone. In contrast, our survey focuses entirely on research that resulted in a practical, online and self-contained implementation on a smart phone.

## 3. Online Activity Recognition

**Figure 1.**
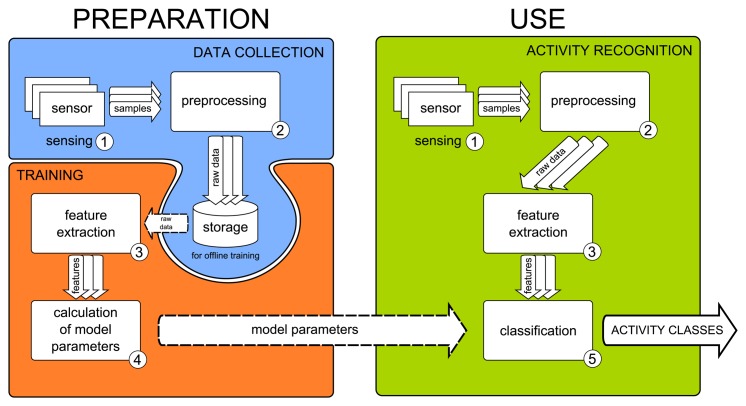
Activity recognition steps.

**Figure 2.**
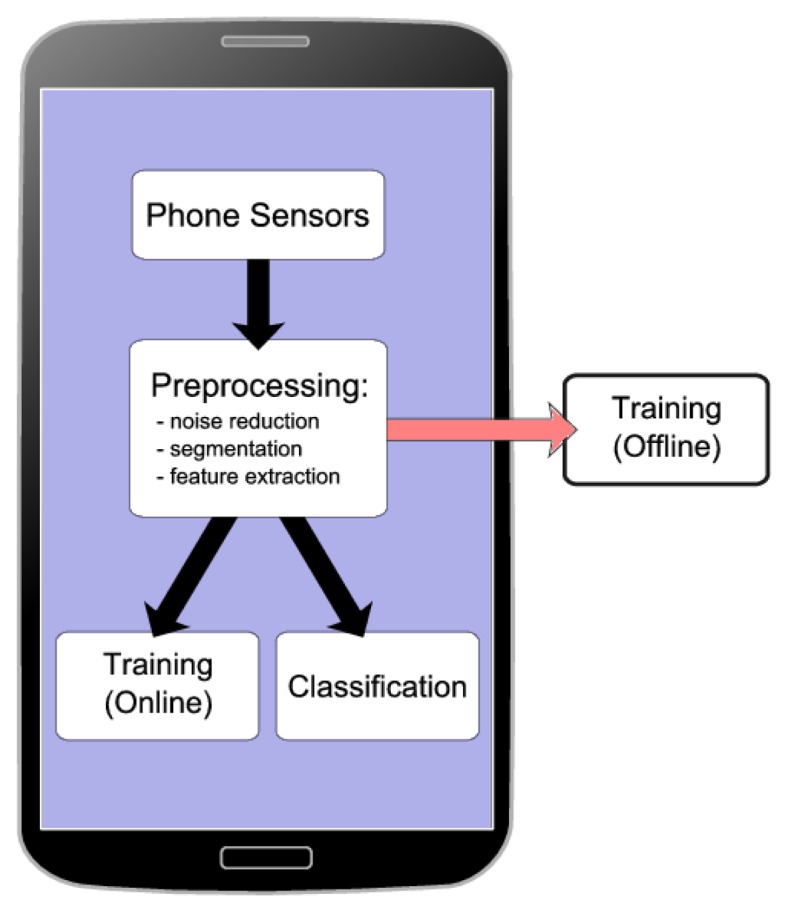
Local approach for activity recognition on mobile phones.

Activity recognition systems consist of mainly four steps: sensing, preprocessing, feature extraction and either training or classification [28]. The steps are shown in Figure 1 and described as follows:

(1) Sensing: In this step, sensor data are collected at a specific sampling rate.
(2) Preprocessing: Subsequently, the collected data are processed in various ways. For example, noise is removed. Then, a windowing or segmentation scheme is applied to it.
(3) Feature extraction: Various data features are extracted from the segmented raw data.
(4) Training: Before an activity recognition system can be used, its classifiers need to be trained. As shown in Figure 1, training is a preparation step to obtain the model parameters for later use in classification. As shown in Figure 2, training can either be offline on a desktop machine or online on the phone itself. If training is performed offline, raw data from example activities is first collected and stored. At a later time, these data are used for obtaining the model parameters, as shown in Figure 1. If training is performed online, the raw data are not stored for later use, but instead directly processed for training. The training step is performed infrequently, and the resulting model parameters are stored for future use in the actual online activity recognition.
(5) Classification: In this final stage, the trained classifiers are used to classify different activities. This step can be done either offline in a machine learning tool, such as WEKA, or online on the mobile phone itself. In this review paper, we are reviewing studies that use online classification.

Mobile phones are being used in two ways for online activity recognition. These two approaches are:

- Client-server approach: In this case, the sensing part is done on the mobile phone, which acts like a client. Then, the collected data are sent to a server or a cloud for further real-time processing, such as preprocessing and classification. The preprocessing step can partially reside on the mobile phone, too. However, the main classification step is performed on a server. This approach is adapted in order to run the computationally-expensive steps on a server, because of the limited resources in a mobile device. For example, in [29], raw data are sent to a server for classification. There are other studies that have done the same [30]. This approach requires an Internet connection at all times for sending sensor data for further processing to a server or a cloud.
- Local approach: In this case, activity recognition steps are done locally on the mobile phone in real time. These steps include data collection, preprocessing and classification. In this approach, information about classified activities and raw data can also be sent to a server for further analysis. However, the main three steps are performed locally. The training can still be done on a desktop machine beforehand or on the phone locally. This approach is shown in Figure 2.

We only consider studies that have followed the local approach, as most of the studies follow this approach. Moreover, our goal is to see the potential of mobile phones in running activity recognition systems locally. It is important to note that there are studies that do more than simple activity recognition, such as in [31]. However, we only mention those parts of these studies that fit the scope of this paper. We discuss these studies in the following aspects:

- Implemented Classification Methods on the Mobile Phones
- Online *vs.* Offline Training for Classification Methods
- Platforms, Phones and Sensors Used in Online Activity Recognition
- Resource Consumption Analysis
- Real-time Assistive Feedback
- Validation of Online Activity Recognition
- Orientation-Independent Activity Recognition
- Position-Independent Activity Recognition
- Fixed and Adaptive Sampling
- Dynamic and Adaptive Sensor Selection
- Performance Evaluation
- Recognized Activities
- Data Features Used for Classification

### 3.1. Implemented Classification Methods on the Mobile Phones

Classification is an important step in the activity recognition process. There are various classifiers that have been implemented on mobile phones in the last few years. The most commonly-used classifiers are decision tree, support vector machine (SVM), K-nearest neighbor (KNN) and naive Bayes. Some of the other implemented classifiers are decision table, rule-based classifier, fuzzy classification, quadratic discriminant analysis (QDA) and neural networks. In some studies, two classifiers are combined in different ways, thereby creating multi-layer or hierarchical classification. For example, decision tree and dynamic hidden Markov model (DHMM) are used in combination in [32]. Studies that show that these classifiers can run on mobile phones are shown in Table 1. A mapping of various classification methods with respect to the studies where they were implemented is given in Table 1. For specific implementation details about these classifiers, readers are referred to the relevant studies, as shown in Table 1.

### 3.2. Online vs. Offline Training for Classification Methods

For classifying test data into various pre-defined classes in supervised classification, the classifiers need to be trained first using training data [28]. In the context of mobile phones, this training can be done in two ways: online and offline.

- Online: the classifiers are trained on the mobile phones in real time.
- Offline: the classifiers are trained beforehand, usually on a desktop machine.

We found that most studies have used the offline method. One of the reasons for doing so was because the training process is computationally expensive. Moreover, it is easy to implement only the classification part on the mobile phone. Only six out of all 30 studies were using online training where classifiers can be trained on mobile phones in real time. We outline all of the studies where an offline and online method has been used in Table 2. It is important to note that all these studies implement real-time classification. In addition to these studies, Google also now provides a real-time activity recognition API [61].

### 3.3. Platforms, Phones and Sensors Used in Online Activity Recognition

Activity recognition systems are implemented on different mobile phone platforms. The most commonly-used among these platforms is Android. However, some studies were conducted using iOS and Symbian. We found only one study with an implementation on the Debian Linux platform using the OpenMoko Neo Freerunner mobile device. All of these studies range from 2008 to 2014. Initially, activity recognition research mainly used the Symbian platform. However, in recent years, Android took over, and most of the studies are now using Android. These studies use different types of mobile phones for their implementations. For example, Nokia N95 is mainly used with the Symbian operating system, various android phones with Android and iPhones with iOS. A detailed description of these phones is given in Table 3. Moreover, the relationship between different studies and their used platforms is given in Table 4.

These studies use different types of motion sensors in the leading role in the activity recognition process. The accelerometer was the dominant sensor in all of these studies. It was used in all studies, in most cases individually, and in a few cases, in combination with other sensors, such as microphone, gyroscope, magnetometer, GPS and pressure sensor. In some cases, these sensors are fused at a raw level, whereas in other cases, at a higher level, depending on the application objective. There are 23 out of 30 studies that used the accelerometer alone. The details about the relationship between the sensors used and their relevant studies are given in Table 5. In this table, A stands for an accelerometer, G for gyroscope, LA for linear acceleration, Mic for microphone, PS for pressure sensor, and M for a magnetometer.

### 3.4. Resource Consumption Analysis

Resource consumption analysis, such as the analysis of battery, CPU and memory usages, is an important aspect of online activity recognition. This is one of the factors in shifting from an offline to an online approach. Such analysis is performed to validate if online recognition systems can be run in real-world settings. However, we found that most of the reported studies are missing this analysis, except a few studies so far, as shown in Table 6.

As shown in Table 6, CPU, memory and battery usage are reported for resource consumption analysis. For the battery consumption, two types of measurements are made. In one case, the amount of time a battery lasted was reported while running online activity recognition systems, as shown in Table 7; while in other cases, the power usage was reported in watt-hours per hour for these systems. Though many studies simply report the number of hours a battery lasts as a resource metric [31,34–36,42,52,59], it has a drawback. Many of these studies use different mobile phones with different battery capacities, so this metric can be misleading. This can be seen in Table 7, where we added the battery capacities to see how different these batteries were. Therefore, watt-hour per hour is a better choice to use for battery usage, as it is independent of the battery capacity. Some of these studies [32,38,43] use both of these metrics. However, this is also not fully platform independent, as these metrics might be affected by other factors in different platforms, for example the CPU speed. The CPU usage was reported in terms of percentages for which the CPU was occupied by recognition process and memory used was reported in MBs (mega bytes). It is difficult to compare these reported values due to their different experimental setups and evaluation methods. For example, it can be seen in Table 7 for battery usage how different various parameters are in these studies. Moreover, these values are presented and discussed in different ways in various studies.

The details about CPU and memory measurements are given in Tables 8 and 9, respectively. These values are hard to compare, because they are reported in different experimental and evaluation setups. To show this, we present some additional information in these tables, such as classifier, platform, sensor and phone. Apart from this information, different data features are used, as shown in Table 3. In some cases, these values only represent specific parts in a complete application, which consists of other parts, too. For example, in [38], these values are only relevant when an accelerometer is being used by an activity classifier, because the activity classifier is a part of an application, which consists of other parts, too, such as a classifier for audio classification. For details on memory and CPU usage in this work, readers are referred to Table 4 in [38].

### 3.5. Real-Time Assistive Feedback

Real-time assistive feedback is an important aspect of the healthcare and other context-aware applications built on top of activity recognition systems to improve people's well-being. However, most of the studies on online activity recognition are missing this feature. There are only two [31,54] out of 30 reviewed studies that provided the capability of real-time feedback for assisting people.

In [54], basic activities are recognized on the mobile device using its accelerometer. These recognition results are sent to a server that used richer context information to give real-time audio feedback to help people with cognitive impairment in doing their daily living activities. They conducted a user study in a smart-home environment in which two users performed various activities, such as cleaning, cooking and watering plants. They were given real-time feedback using their mobile phones whenever they made a mistake in performing these activities. The authors evaluate the feedback's timing and perceived helpfulness for the users based on their experiences.

In [31], a user study is conducted with one user for one week, where the authors evaluated the effectiveness of their real-time feedback mechanism. They use an animated display on the mobile phone lock-screen and wall-paper, so a user can see the well-being results every time he or she interacts with his or her mobile phone. The well-being is measured in scores such as sleep, activity and social interaction level. These values are represented by an animated turtle, clown fish and a school of fish, respectively. A user behavior's is reflected by these animated entities, thereby giving him or her real-time implicit feedback if these levels are low. For example, if a user is not active, the clown fish remains inactive or moves slowly on the screen. The fish movements become stronger as the user activity level goes up. The relationship between user well-being and these animated entities is explained in detail in [31].

Besides the above two studies, in [38], the authors refer to the feedback as near to real-time. However, this feedback is more in the social context rather than personal by sharing information with a group of friends. The feedback about the presence information (dancing at party) of a user is posted in almost real-time on social networking sites, such as Facebook, for other users.

### 3.6. Validation of Online Activity Recognition

To validate the online activity recognition systems, they need to be evaluated in a testing scenario. For this purpose, a number of participants is needed to use mobile phones running these systems for a certain amount of time, which we refer to as the testing duration. Many of the studies are limited in the evaluation part, since the testing of these systems was not performed thoroughly. In some cases, both the number of subjects and the testing duration are not mentioned in the publications, and in other cases, the number of subjects is mentioned, but the testing duration is not. There are other studies where the testing is done for less than an hour. For example, 36 min in [50], 15 min in [34] and 8 min in [49]. The testing scenarios for all of these studies are summarized in Table 10, where NA stands for not available. For some studies, there was no information available on how they tested their implemented systems [33,35,36,45,51,53,55,60].

### 3.7. Orientation-Independent Activity Recognition

Activity recognition results are sensitive to some of the sensors' orientation changes, such as the accelerometer and gyroscope. Most of the reviewed studies in this paper use an accelerometer, as shown in Table 5. With the lack of orientation independence, users are required to place the mobile device in a specific orientation, which limits their freedom to use the mobile devices. Therefore, in order to have a practical activity recognition solution, it should be orientation independent. The orientation independence can be achieved mainly in two ways [36,39]:

- Using orientation-independent features: To counter mobile phone orientation changes, usually orientation-independent features are used. For example, features are calculated based on accelerometer magnitude instead of their individual three-axis, because the accelerometer magnitude is less sensitive to orientation changes [39].
- Using signal transformation: In this method, usually, various types of signal transformations are used. For example, in most cases, the coordinate system of the mobile device is transformed into a global Earth coordinate system for countering orientation changes [36].

We divide these studies into two categories. One is where it is explicitly mentioned that their proposed solutions are orientation independent, and the second one is where such information is not mentioned. We found nine studies where orientation independence is considered and mentioned. In Table 11, these studies are listed with their approaches to counter orientation changes. In this table, OI-F stands for orientation-independent features and OI-ST means orientation-independent signal transformation.

In [32], for example, the standard deviation of accelerometer magnitude is used as an orientation-independent data feature. In this study, they use the orientation-independent features method, and accelerometer magnitude is one of the ways to counter orientation changes. Similarly, in [51], features are calculated based on accelerometer magnitude. Though they do not talk explicitly about orientation independence, it can be considered an orientation-independent approach. Moreover, orientation-independent features are used in [39,52]. On the other hand, the signal transformation approach is used in [33,36,53], where the mobile phone coordinate system is converted into a global Earth coordinate system. In [59], both of these approaches are used in combination. First, the signal transformation is used to convert the mobile coordinate system into a global coordinate system, and then, orientation-independent features are extracted from the transformed data. In [60], the authors use a novel training structure to counter the orientation changes in real time using fuzzy classification.

### 3.8. Position-Independent Activity Recognition

One of the challenges in activity recognition using mobile phones is that motion sensors are sensitive to body position. In most studies, the position of the mobile phones is kept fixed, because any changes in position may result in a loss of recognition performance. There are a few studies among the reviewed ones that try to solve this problem with different methods. We divide these methods into four types, which are described as follows:

- Method 1: In some studies, a generalized classifier is trained using data from all relevant positions. Moreover, position-/orientation-independent features are also used in combination with such a generalized classifier.
- Method 2: A generalized classifier is trained using data from all relevant positions, and discriminant analysis is used after feature extraction.
- Method 3: In this method, different sub-classes are used for different body positions. For example, walking with a phone in the pocket position is considered a different class than walking with a phone in the hand. Therefore, for a classifier, these two are different classes, but at a higher level, this is one activity, such as walking.
- Method 4: In this method, a separate classifier is trained for each position. In the real-time recognition process, the system first estimates the position and then uses the classifier trained for that specific position.

In Table 12, we outline the studies that have implemented position-independent activity recognition. These studies are not completely position-independent, however; they try to minimize the effects of changing positions. NA stands for not available in Table 12. Studies with NA did not mention explicitly whether they are position dependent or not. However, we believe that they can be considered as position dependent, because position independence is an important contribution and would likely have been explicitly highlighted in these studies.

### 3.9. Dynamic and Adaptive Sensor Selection

Dynamic and adaptive sensor selection can be used to improve battery life. This means that various sensors are turned on and off in real time in an adaptive way for energy-efficient activity recognition. In our analysis, we found two out of 30 studies [41,42] that considered such a dynamic and adaptive approach for sensor selection in their implementations. In [41], GPS is used in combination with an accelerometer to detect different activities, only when the user is performing physical activities outdoors. However, it intelligently disables the GPS as soon as a user enters a building. GPS is not useful indoors for recognizing indoor activities, and the accelerometer can provide reasonable recognition accuracy. In [42], the authors present a framework for energy-efficient mobile sensing. It has the ability to turn on and off various sensors in an adaptive way for energy efficiency.

### 3.10. Fixed and Adaptive Sampling

The sampling rate plays an important role in the activity recognition process. The choice of a suitable sampling rate is a design decision that can be affected by factors, such as the available resources, desirable accuracy and the type of data features being used for activity recognition. For example, if only frequency domain features are used for recognizing human activities, then the sampling rate should be high enough to capture all of the relevant frequencies for human movements. According to the authors in [62–64], all measured body movements are contained within frequency components below 20 Hz. Moreover, in [65], the authors showed that human activity frequencies lie between 0 and 20 Hz and that 98% of the FFT (fast fourier transform) amplitude is contained below 10 Hz. Based on these analyses, the sampling rate should be chosen accordingly (using the Nyquist formula), so that all relevant frequencies are captured. A higher sampling rate can increase the accuracy to a certain extent, but it will cause higher battery consumption and *vice versa*. In our survey, we found that various sampling rates were used, of which 50, 32 and 20 Hz were the most common, as shown in Table 13. Overall, we observe a range from 2 Hz to 125 Hz with reasonable reported accuracies in these studies. Though various rates within this range are suitable for human activity recognition, there is a trade-off between accuracy and resource consumption, as we discuss in Section 4. The details about these rates are given in Table 13. Moreover, the relationship between these sampling rates and battery consumption is given Table 8, and a more detailed description is in Table 14. It is important to note that some studies did not mention which sampling frequencies they used in their implemented systems. These are classified as NA (not available) in Table 13.

Most studies in Table 13 use a fixed sampling rate. There are only two studies that used adaptive sampling for energy efficiency. In [43], the authors use adaptive sampling and duty cycling techniques with the accelerometer sensor to improve the battery life, while maintaining a reasonable recognition performance. In [36], the authors use an accelerometer and GPS, whereas GPS is sampled in an adaptive way for energy efficiency.

### 3.11. Performance Evaluation

For the evaluation of an online activity recognition system, there is the need for a performance metric. There are many performance metrics that can be used for the evaluation of activity recognition systems, such as accuracy, precision, false positives, false negatives and the F-measure. The choice of a specific performance metric or a combination of different performance metrics depends on the type of application and its performance requirements [28,66]. The details of different performance metrics for activity recognition can be found in [28,66]. Most of the studies in our review use accuracy as their main performance metric, except [41,45,55], where no results are reported for the implemented systems. In some studies, other performance metrics have been used alongside accuracy, such as precision, ROC area, false positive rate and the F-measure. For example, in [33], the authors use accuracy, false positives, precision, ROC area and the F-measure to compare the recognition performance of a machine learning algorithm for various physical activities. Moreover, in [34,36], the authors use accuracy and precision for performance evaluation.

In this survey, we do not include the accuracies reported in all reviewed studies, because it is difficult to compare them, due to their different experimental setups and different set of activities, as shown in Tables 14 and 3.

### 3.12. Recognized Activities

In all of these studies, different types of physical activities are recognized. The most common are walking, running, biking, jogging, still (stationary), walking upstairs and walking downstairs. Some of the examples of other activities used in these studies are driving a car, jumping, riding a bus, vacuuming and using the elevator. A detailed description of these activities and their relevant studies are given in Tables 14 and 3. These two tables show which type of experimental setups, classifiers, data features, sampling rates, *etc.*, are used for recognizing various sets of different activities.

The set of activities that are being recognized play an important role in the design decisions of an activity recognition system. For example, it can help developers or researchers to make different choices, such as which sensors, features or classifiers to use. For example, for a small set of simple activities, it is better to use a single accelerometer and not a fusion of multiple sensors or a very high sampling rate. We have shown previously [10] that a gyroscope is unable to differentiate between the sitting and the standing activity. Therefore, using only a gyroscope when recognizing a set of activities that contains both sitting and standing activities is not a good idea. On the other hand, the accelerometer performs poorly with walking upstairs and downstairs. However, the combination of an accelerometer and gyroscope performs better in recognizing walking upstairs and walking downstairs activities. Therefore, it is recommended to use a combination of gyroscope and accelerometer when recognizing a set of activities that contains both walking upstairs and downstairs activities. Moreover, a barometer can be combined with inertial sensors to recognize a set of activities that involves walking upstairs and downstairs in a better way. Its independence of orientation and position makes it very useful. The usefulness of fusing a barometer with the accelerometer for better activity recognition has been shown in a few studies [67,68]. For example, in [68], the authors show that the use of a barometer increases the recognition performance for walking upstairs and downstairs by up to 20%. Higher accuracies have also been reported for climbing up and down activities in [67] using a combination of the barometer and accelerometer.

### 3.13. Data Features Used for Classification

In the preprocessing phase, various features are extracted from the sensor data. These features are used in the training and testing of classification methods. There are two main types of data features: time and frequency domain features. Both of these types of features are used in online activity recognition. However, time domain features are more commonly used. For the time domain, the most commonly-used features are the mean, variance (VAR) and standard deviation (SD). In terms of computation costs, the time domain features are cheaper than the frequency domain features, because of the extra Fourier transform calculation [69]. A detailed discussion about the memory and processing requirements for these features can be found in [69], where the authors describe the complexities of these features, as well as their suitability for mobile devices.

A description of various studies and their relevant features used for online activity recognition is given in Table 3. This table shows the list of features that are implemented on mobile devices.

## 4. Discussion

We presented various aspects of the online activity recognition studies in the previous section. It can be seen now that it is difficult to compare all of these studies in terms of recognition performance and resource consumption, because they use different experimental setups, classifiers and evaluation methods, as shown in Tables 1, 4, 5, 13 and 3. Moreover, different types of activities are recognized in these studies, as shown in Table 3, which makes comparing them difficult. However, here, we present an overview of some general trends among these studies. Moreover, we highlight which aspects can be improved.

Most of the studies have implemented one classification method. It is difficult to compare classification methods from different studies for their accuracy and resource consumption, because of their different experimental setups, as shown in Tables 1, 4, 5, 13 and 3. Therefore, it is important to compare multiple classification methods in a standard experimental setup, where their relative suitability for mobile phones can be reported. This can help in making design decisions for other researchers to implement a specific classifier according to their needs in the future. There are very few studies that have implemented multiple classifiers for comparison purposes, and there is still room for further research. For example, two classifiers in [36,50] and three in [37] are implemented on mobile phones and are compared for their evaluation results.

We also found that most of the studies are missing resource consumption analysis, such as CPU, memory and battery usage, as is shown in Table 6. For the online activity recognition, such an analysis is an important factor in determining the feasibility of its implementation on a mobile phone. So far, comparative studies have been done offline, where different classification methods are compared in different simulation setups based on the accuracy only. We believe that is not a fair comparison. In order to report a classification method or a set of features as most suitable for activity recognition on mobile phones, it is important to consider the trade-off between accuracy and resource consumption. Only then can it be decided which feature sets and classification methods perform better than others and at what cost.

We also noticed that many studies have no proper evaluation for activity recognition systems implemented on mobile phones. This is clear from Table 10. In some cases, both the testing duration and the number of subjects involved is not mentioned. In other cases, the number of subjects is mentioned, but not the testing duration. We noticed that in many cases, most of the performance analysis is done offline, and then, the best available feature sets and classification methods were implemented online, as shown in Tables 1 and 3. However, the online evaluation results are not available in their publications.

In some studies, different sensors are combined, but their individual contribution to the overall performance and their resource consumption costs are not evaluated, such as in [37,47]. This makes it unclear if such a fusion is actually helpful. Once it is clear to what extent an additional sensor improves the overall performance and at what cost in terms of resource consumption, it is useful for deciding when to fuse various sensors in the activity recognition process. Moreover, most of these studies use a static combination of sensors. There is also the need for more work on the adaptive selection of sensors in a dynamic way, which can result in energy-efficient solutions. There were only two studies out of 30 that used dynamic and adaptive sensor selection in their implementations. Besides the static selection of sensors, in most cases, the sampling rates were also fixed. This area can be improved by investigating adaptive sampling techniques for better energy usages, while maintaining an acceptable level of recognition performance.

The selection of suitable features is also an important factor towards practical online activity recognition. The exact number and type of features is a design decision. For this purpose, it is important to see how much the addition of a feature improves the overall performance and how much resource consumption it causes. In some studies, as shown in Table 3, many features are used without evaluating their individual contributions, for example, especially in [39,52]. One solution could be to start with a simple possible set of features and then see the improvements that are caused by additional features.

The lack of personalization in these studies is another important aspect that needs further research. Most studies use classification methods that are trained offline, thereby making the training process static. These systems may not adapt to new users. Some users walk very slowly compared to others. The same behavior can be seen in other activities, like using stairs, running and biking. The performance of such statically-trained classifiers is dependent on the type of users on which they are being tested and can affect the recognition performance. This problem can be solved by providing an online training option in these implementations. With the help of such an option, users can adapt these classifiers according to their own needs, making it more personalized. Moreover, some activities can change in different situations due to environmental factors. For example, walking upstairs and downstairs activities depend on the type and design of stairs. A classifier trained for one type of stairs may not perform well on other types of stairs. We also need the online training ability in these kinds of situations to make them more personalized. We showed that most of the studies are using fixed position and orientation for online activity recognition, as shown in Tables 12 and 11. However, this limits users' freedom to use their phones in different ways, which makes such solutions less practical and less attractive for them. In order to move towards more practical and attractive solutions, these two aspects should be further explored with an actual implementation.

Though activity recognition systems can be used in many areas, they have attracted special attention in the healthcare domain for developing well-being applications. For such applications, one of the important aspects is real-time assistive feedback, which can be used to improve human cognition in many ways. Unfortunately, we found only two studies out of 30 that have explored this aspect. This shows room for further research in this area on how to interact with users and how to use assistive feedback in an effective way. Inspirations can be taken from the studies where the mobile phone is not used as the main platform for sensing and classification, but used as a feedback device or interface. For example, the authors in [70] use a dedicated activity recognition device for sensing, but use a mobile phone as a feedback device. They developed an interactive garden wallpaper for the mobile phone, which is used to motivate the user to be physically active. In [71], the light is used as feedback for improving physical activity. However, this has been done on a wrist-worn device. Still, this idea can be used with mobile phones and needs further exploration. For real-time assistive feedback, mobile phones can be used in various ways, such as utilizing the text messaging, audio and mobile phone display. For example, in [72], the authors use real-time musical feedback to motivate users to achieve their exercise goals. We do not go into detail on feedback applications, as those can be designed in different ways, for example interactive games [73]. However, details of the interaction with users for assistive feedback using mobile phones can be found in [16,27,74].

One of the important points that we observed in these studies is that in some cases, important information is not mentioned explicitly. For example, sampling rates are not mentioned in some studies, as shown in Table 13. In some cases, other details are missing, such as whether the provided solutions are position and orientation independent. There were also cases where the number of subject or the duration of the evaluation was not mentioned, as shown in Table 10. This information plays an important role in comparative research on this topic and for reproducing this work. Therefore, this information should be mentioned in papers related to online activity recognition.

Though there has been significant work done in online activity recognition on mobile phones, we believe it is still in its infancy. That is one of the reasons why we see some of these limitations in the current studies, which provide us with room for future work. Therefore, we present the following recommendations for future studies. These recommendations will help in comparing these studies with each other and with future studies in a better way. Moreover, they can be used for making design decisions in implementing such systems.

- There should be an analysis of resource consumption, which includes memory, CPU and, most importantly, the battery usage. This analysis should explore the trade-off between the resource usage and the recognition accuracy.
  – For the comparison of multiple alternative classifiers and preprocessing steps, their evaluation should be performed online on the mobile phone rather than offline. Instead of implementing one classifier, multiple classifiers should be implemented in the same experimental setup for comparison purposes to know their relative suitability for mobile phones in the context of accuracy and resource consumption. This can provide us with a benchmark for future studies.
- There should be a proper evaluation of the implemented system on the mobile phone, which means that the system should be tested for a reasonable amount of time by a reasonable number of subjects. It can be further researched what the reasonable amount of time and number of subjects are. Moreover, this information should be explicitly described in the published work.
- The online activity recognition system should support personalization, so that users can train such a system online according to their needs. This can be achieved by moving from offline training towards online training of classification methods on the mobile phones.
- The research should be reproducible. This means that the implementation details of the employed classification algorithms and preprocessing steps should be explicitly described. Different implementations of the same algorithm can lead to different evaluation results, which is important for comparison purposes.
- For energy-efficient online activity recognition, adaptive sampling methods and duty cycling in real time should be explored. Moreover, dynamic sensor selection for energy-efficient activity recognition should be further studied in practical implementations.
- Real-time assistive feedback should be explored further on top of the online activity recognition systems to show the practicality of such systems in well-being and other context-aware applications.
- Position and orientation independence should be further explored for practical activity recognition. Moreover, it should be explicitly mentioned if these properties are considered in the relevant implementations of such systems in future studies.
- In the activity recognition process, blind fusion of mobile phone sensors should be avoided. Where multiple sensors are combined to achieve better recognition accuracy, each additional sensor should be evaluated for its resource consumption and its contribution towards the overall performance.
- Feature selection is an important design decision. Light-weight features that are suitable for running on mobile phones should be selected. Moreover, the contributions, both towards overall performance and resource consumption, of various features need to be evaluated to avoid the blind addition of redundant features.

In the current work, it is not possible to compare different methods because of the different experimental setups. Therefore, in future studies, it is important to compare multiple feature sets and classification methods in similar experimental settings. This can help with setting a benchmark on which further studies can be based.

## 5. Conclusions

In this paper, we reviewed the work done so far on online physical activity recognition using mobile phones. We consider studies that use only mobile phone sensors and that do the classification locally on mobile phones in real time. Moreover, these studies consider recognizing multiple activities. We found such 30 studies that have implemented online activity recognition on mobile phones. We discuss various aspects of these studies and their limitations. Some of these aspects included their experimental setups, evaluation methods, real-time assistive feedback, position and orientation independence, adaptive sensor selection and sampling and resource consumption analysis. Moreover, we discussed the areas that need further improvements. Finally, we presented various recommendations for conducting future studies for online activity recognition on mobile phones.

## Figures and Tables

**Table 1.** Implemented classifiers on mobile phones for online activity recognition.

**Implemented Classifiers**	**Relevant Studies**	**Total Relevant Studies**
Decision Tree	[33–43]	11
SVM	[44–49]	6
KNN	(Clustered KNN [50]) [51–53]	5
Naive Bayes	[31,37,50,54,55]	4
Multi-layer Classifiers	(Decision tree, dynamic hidden Markov model (DHMM) [32]) (SVM, K-medoids clustering [56]) (Decision tree, probabilistic neutral network (PNN) [57])	3
Probabilistic Neural Networks	[58]	1
Rule-based Classifier	[59]	1
Quadratic Discriminant Analysis	[52]	1
Decision Table	[37]	1
Fuzzy Classification	[60]	1

**Table 2.** Training process on mobile phones (online *vs.* offline).

**Training Process (Online *vs.* Offline)**	**Relevant Studies**	**Total Relevant Studies**
Offline	[31–43,46,47,51–54,56–60]	24
Online	[44,45,48–50,55]	6

**Table 3.** Phones, activities and data features used in online activity recognition.

**Study**	**Activities**	**Data Features**	**Phone**
[38]	A1, A2, A3, A5	mean, SD, number of peaks	Nokia N95
[41]	A1, A5, A8, A9, A10	A's mean, VAR, FFT coefficients and GPS speed	Android Phone, Nokia N95
[42]	A1, A5, A8, A11	SD (based on accelerometer magnitude)	Nokia N95
[60]	A1, A2, A3, A6, A9, A16, A17, phone in hand, typing text messages; talking on the phone	mean, VAR	OpenMoko Neo Freerunner
[51]	A1, A5, A6, A7, A12, A17, Idle	Fundamental frequency, average acceleration, max and min amplitude (based on accelerometer magnitude)	Motorola Droid
[36]	A1, A5, A8, A9, A11	mean, VAR, mean crossing rate, spectrum peak, sub-band energy, sub-band energy ratio, spectral entropy	Nokia N95, iPhone
[32]	A1, A5, A8, A9, A11	A's VAR, DFFTcomponents and GPS speed	Nokia N95
[45]	A1, A4, A5, A12	similarity score using geometric template matching algorithm	Android phones
[31]	A1, A5, A8, A10	mean, VAR	Android Nexus One
[54]	A1, A2, A3, A5, A6	mean, root mean square, difference between max and min values	Android phones
[55]	Different physical activities	maximum and minimum euclidean norm	ZTE Blade
[59]	A1, A2, A3, A4, A5, A9	signal magnitude, coefficient of variance, counts per minute	Samsung Galaxy S
[50]	A1, A2, A3, A5	mean, min, max, SD	Samsung Galaxy Gio
[34]	A1, A3, A5	mean, VAR, SD, correlation between axes, inter-quartile range, mean absolute deviation, root mean square and energy	HTC Evo 4G
[35]	A1, A2, A3, A5, A6, A7, A9, A10, A12, A16 (prone, supine)	mean (axis, magnitude), SD (axis, magnitude), tilt, linear regression coefficients, wavelet coefficients	HTC G11, Samsung i909
[48]	A1, A5, A15, A16, WD, IR, BT, HD, FTT, BRD, unknown	A's VAR, MFCC (Mel-frequency cepstral coefficient), RMS (root mean square), ZCR (zero-crossing rate) as acoustic features.	Android phone
[40]	A1, A2, A4, A6, A7	peak, SD/mean, FFT energy	HTC Hero
[52]	A1, A2, A3, A5, A9, A10	21 features, including mean, SD, min, max, 5 different percentiles and observations below/above these percentiles	Samsung Galaxy Mini, Nokia N8
[49]	A1, A1 (power), A4, A8	For details, refer to [49]	iPhone
[43]	A1 (slow), A2, A3 (relax, normal), A7, A13, A14	Mean, VAR, magnitude, covariance, FFT energy and entropy	Nokia N95, Samsung Galaxy S2
[44]	A1, A2, A3, A6, A7, A16	For details, refer to [44]	Samsung Galaxy S2
[33]	A1, A5, A6, A7, A8, A9, A10	9 features based on the auto-correlation function of accelerometer signals	Samsung Galaxy Y
[58]	A1, A2, A5, A6, A7, A12	Auto-regressive coefficients	LG Nexus 4
[47]	A1, A5, A6, A7, A8	SD and auto-regressive fitting of y-axis, correlation of x, y, z, signal magnitude area, mean, SD and skewness of the pitch	Android smartphone
[37]	A1(slow, normal, rush), A2, A3, A5	Mean, VAR, zero crossing rate, 75th percentile, correlation, inter-quartile, signal energy, power spectrum centroid, FFT energy, frequency-domain entropy	Google Nexus S
[39]	A1, A2, A3, A5, A9, A10, A17	SD, min, max, the remainder between percentiles (10, 25, 75, 90), median, the sum, square sum and number of crossings of values above or below the percentile (10, 25, 75 and 90)	Nokia N8, Samsung Galaxy Mini
[53]	A1, A2, A3, A5, A12, A16	mean, SD	iPhone 4S
[56]	A1, A4, A6, A7, A9	time gap peaks, mean, SD, A's energy, Hjorth mobility and complexity	HTC Nexus
[57]	A1, A4, A6, A7, A8, A9, A13, A14	Average period, VAR, average energy, binned distribution for each axis and correlation between y and z	Samsung Nexus S
[46]	A1, A5, A1/A5 on treadmill, A6, A7, A9, A10, A11, A12, A13, A14, A15, idle (A2/A3), watching TV	mean, SD, correlation, signal magnitude area, auto-regressive and moving average coefficients for A; altitude difference for pressure sensor; mean, VAR, min and max for audio sensor	LG NEXUS 4

Activities: walking, A1; standing, A2; sitting, A3; jogging, A4; running, A5; walking upstairs, A6; walking downstairs, A7; still, A8; biking, A9; driving a car, A10; in vehicle, A11; jumping, A12; using elevator up, A13; using elevator down, A14; vacuuming, A15; laying, A16; phone on table/detached, A17; washing dishes, WD; ironing, IR; brushing teeth, BT; hair drying, HD; flushing the toilet, FTT; boarding, BD; unknown

**Table 4.** Mobile phone platforms used for online activity recognition.

**Mobile Phone Platforms**	**Relevant Studies**	**Total Relevant Studies**
Android	[31,33–35,37,39–41,43–48,50–52,54–59]	23
Symbian	[32,36,38,39,41–43,52]	8
iOS	[36,49,53]	3
Debian Linux	[60]	1

**Table 5.** Sensors used for online activity recognition. A, accelerometer; G, gyroscope; M, magnetometer.

**Mobile Phone Sensors**	**Relevant Studies**
A	[31,33–36,38–40,42–45,49–56,58–60]
A, M	[57]
A, Mic	[48]
A, GPS	[32,41]
A, G, M	[47]
A, PS, Mic	[46]
A, G, M, Gravity Sensor, LA, Orientation Sensor	[37]

**Table 6.** Studies with resource consumption analysis.

**Resources (Performance Metric)**	**Relevant Studies**	**Total Relevant Studies**
CPU (percentage)	[31,32,36,38,39,50,52,60]	8
Memory (MBs)	[31,32,37,38,50,56]	6
Battery (hours or watt-hours per hour)	[31,32,34–36,38,42–44,52,56,59]	11

**Table 7.** Details of battery usage analysis.

**Study**	**Implemented Classifiers**	**Sensors**	**Platform**	**Phone**	**Battery Lifetime (h)**	**Battery Capacity (mAh)**	**Sampling Rates**
Miluzzo *et al.* [38]	Decision tree	A	Symbian	Nokia N95	6	950	Various rates
Wang *et al.* [42]	Decision tree	A	Symbian	Nokia N95	11.3	950	NA
Reddy *et al.* [32]	Decision tree + DHMM	A, GPS	Symbian	Nokia N95	8.3	950	32 Hz
Lu *et al.* [36]	Decision tree	A	Symbian	Nokia N95	16	950	32 Hz
Lane *et al.* [31]	Naive Bayes	A	Android	Android Nexus One	15	1400	NA
Guiry *et al.* [59]	Rule-based classifier	A	Android	Samsung Galaxy S	6–8	1500	90 Hz
Siirtola, [52]	KNN, QDA (quadratic discriminant analysis)	A	Android	Samsung Galaxy Mini, Nokia N8	24	1200, 1200	40 Hz
Lara *et al.* [34]	Decision tree	A	Symbian, Android	HTC Evo 4G	12.5	1500	50 Hz
Liang *et al.* [35]	Hierarchical recognition scheme using decision tree	A	Android	HTC G11, Samsung Galaxy S2	7, 8, 10	1450, 1650	20, 10, 2 Hz

**Table 8.** Studies with CPU usage analysis.

**Study**	**Implemented Classifiers**	**Platform**	**Phone**	**Sensors**	**CPU Usage %**
Miluzzo *et al.* [38]	Decision tree	Symbian	Nokia N95	A	31
Reddy *et al.* [32]	Decision tree + DHMM	Symbian	Nokia N95	A, GPS	4.72
Lu *et al.*[36]	Decision tree	Symbian, iOS	Nokia N95, iPhone	A	iPhone (0.9–3.7), Nokia N95 (1–3)
Berchtold *et al.* [60]	Fuzzy classification	Debian Linux	OpenMoko Neo Freerunner	A	3.3
Lane *et al.* [31]	Naive Bayes	Android	Android Nexus One	A	11
Siirtola [52]	QDA	Symbian, Android	Samsung Galaxy Mini, Nokia N8	A	5
Kose *et al.* [50]	Naive Bayes, KNN clustered	Android	Samsung Galaxy Gio	A	42 (Naive), 29 (KNN clustered)
Siirtola and Roning. [39]	Decision tree	Symbian	Nokia N8	A	15

**Table 9.** Studies with memory usage analysis.

**Study**	**Implemented Classifiers**	**Platform**	**Phone**	**Sensors**	**Memory Usage (MB)**
Miluzzo *et al.* [38]	Decision tree	Symbian	Nokia N95	A	34
Reddy *et al.* [32]	Decision tree + DHMM	Symbian	Nokia N95	A, GPS	29.64
Lane *et al.* [31]	Naive Bayes	Android	Android Nexus One	A	14.74
Kose *et al.* [50]	Naive Bayes, KNN clustered	Android	Samsung Galaxy Gio	A	12.6 (naive Bayes), 21.9 (KNN clustered)
Martin *et al.* [37]	Decision table, naive Bayes, decision tree	Android	Google Nexus S	A, M, G, linear acceleration, gravity	16.5 (decision table), 0.00146 (naive Bayes), 0.8376 (decision tree)

**Table 10.** Evaluation of online activity recognition systems.

**Testing Duration**	**[Relevant Study] (Number of Subjects)**
6–8 weeks by 6 users with Nokia N95/6 days by 2 users with Samsung Galaxy S2	[43] (8)
4 weeks	[32] (1)
1 week	[38] (8), [31] (5), [58] (10)
2 days	[42] (10)
1 day	[37] (2)
Less than an hour	[49] (2), [50] (5), [34] (2), [57] (5)
NA	[47,54,56] (2), [59] (6), [40] (5), [52] (12), [48] (21), [39] (1), [46] (8)
NA	[33,35,36,41,44,45,51,53,55,60] (NA)

**Table 11.** Studies with orientation-independent activity recognition. OI-F, orientation-independent features; OI-ST orientation-independent signal transformation.

**Study**	**Methods Used for Orientation-Independent Online Activity Recognition**	**Used Sensors**
[32]	OI-F	A, GPS
[51]	OI-F	A
[36]	OI-ST	A
[60]	OI-Training	A
[59]	OI-ST + OI-F	A
[52]	OI-F	A
[33]	OI-ST	A
[39]	OI-F	A
[53]	OI-ST	A

**Table 12.** Position-independent activity recognition.

**Position-Independence**	**Relevant Studies**	**Total Relevant Studies**
Yes (Method 1)	[32,33,39,42,53]	5
Yes (Method 2)	[46,58]	2
Yes (Method 3)	[36,60]	2
Yes (Method 4)	[37]	1
No	[31,35,38,40,44,45,47–52,54,56,59]	15
NA	[34,41,43,55,57]	5

**Table 13.** Sampling rates used in online activity recognition.

**Sampling Rate (Hz)**	**Relevant Studies**
50	[34,40,43,44,46,47,49,53,57]
20	[35,48,50,54,58]
32	[32,36,41,56]
100	[43,50,60]
40	[39,52]
10	[35,50]
125	[51]
90	[59]
16, 5	[43]
8	[33]
6.25	[37]
2	[35]
NA	[31,38,42,45,55]

**Table 14.** Detailed comparison of all 30 studies. O-Ind: Orientation Independence; Pos-Ind: Position Independence.

**Study**	**Activities**	**Classifiers**	**Platform**	**Sensors**	**Sampling Rate (Hz)**	**Battery Analysis**	**RAM Analysis**	**CPU Analysis**	**O-Ind**	**Pos-Ind Training**	
[38]	A1, A2, A3, A5	Decision tree	Symbian	A	NA	yes	yes	yes	no	no	offline
[41]	A1, A5, A8, A9, A10	Decision tree	Symbian, Android	A, GPS	32	no	no	no	no	NA	offline
[42]	A1, A5, A8, A11	Decision tree	Symbian	A	NA	yes	no	no	no	yes	offline
[60]	A1, A2, A3, A6, A9, A16, A17, phone in hand, typing text messages talking on phone	Fuzzy classification	Debian Linux	A	100	no	no	yes	yes	yes	offline
[51]	A1, A5, A6, A7, A12, A17, Idle	KNN classifier	Android	A	125	no	no	no	yes	no	offline
[36]	A1, A5, A8, A9, A11	Decision tree	Symbian, iOS	A	32	yes	no	yes	yes	yes	offline
[32]	A1, A5, A8, A9, A11	Decision tree + DHMM	Symbian	A, GPS	32	yes	yes	yes	yes	yes	offline
[45]	A1, A4, A5, A12	SVM	Android	A	NA	no	no	no	no	no	online
[31]	A1, A5, A8, A10	Naive Bayes	Android	A	NA	yes	yes	yes	no	no	offline
[54]	A1, A2, A3, A5, A6	Naive Bayes	Android	A	20	no	no	no	no	no	offline
[55]	Different physical activities	Naive Bayes	Android	A	NA	no	no	no	no	NA	online
[59]	A1, A2, A3, A4, A5, A9	Custom classifier	Android	A	90	yes	no	no	yes	no	offline
[50]	A1, A2, A3, A5	Naive Bayes, KNN clustered	Android	A	10, 20, 100	no	yes	yes	no	no	offline
[34]	A1, A3, A5	Decision tree	Android	A	50	yes	no	no	no	NA	offline
[35]	A1, A2, A3, A5, A6, A7, A9, A10, A12, A16 (prone, supine)	Hierarchical decision tree	Android	A	2, 10, 20	yes	no	no	no	no	online
[48]	A1, A5, A15, A16, WD, IR, BT, HD, FTT, BRD, unknown	SVM	Android	A, audio (mic)	20 (A), 16 k (mic)	no	no	no	no	no	offline
[40]	A1, A2, A4, A6, A7	Decision tree	Android	A	50	no	no	no	no	no	online
[52]	A1, A2, A3, A5, A9, A10	KNN, QDA	Symbian, Android	A	40	yes	no	yes	yes	no	offline
[49]	A1, A1 (power), A4, A8	SVM	iOS	A	50	no	no	no	no	no	offline
[43]	A1 (slow), A2, A3 (relax, normal), A7, A13, A14	Decision tree	Symbian, Android	A	5, 16, 50, 100	yes	no	no	no	NA	online
[44]	A1, A2, A3, A6, A7, A16	SVM	Android	A	50	yes	no	no	no	no	online
[33]	A1, A5, A6, A7, A8, A9, A10	Decision tree	Android	A	8	no	no	no	yes	yes	offline
[58]	A1, A2, A5, A6, A7, A12	PNN	Android	A	20	no	no	no	no	yes	offline
[47]	A1, A5, A6, A7, A8	Hierarchical SVM	Android	A,G,M	50	no	no	no	no	no	offline
[37]	A1 (slow, normal, rush), A2, A3, A5	Decision tree, naive Bayes, decision table	Android	A, M, G, LA, gravity	At least 6.25	no	yes	no	no	yes	offline
[39]	A1, A2, A3, A5, A9, A10, A17	Decision tree	Symbian, Android	A	40	no	no	yes	yes	yes	offline
[53]	A1, A2, A3, A5, A12, A16	KNN	iOS	A	50	no	no	no	yes	yes	offline
[56]	A1, A4, A6, A7, A9	SVM + K-medoids clustering	Android	A	32	yes	yes	no	no	no	offline
[57]	A1, A4, A6, A7, A8, A9, A13, A14	Decision tree + PNN	Android	A,M	50	no	no	no	no	NA	offline
[46]	A1, A5, A1/A5 on treadmill, A6, A7, A9, A10, A11, A12, A13, A14, A15, idle (A2/A3), watching TV	SVM	Android	A, Pressure (ps), audio (mic)	50 (A, PS), 800 (Mic)	no	no	no	no	yes	offline

Activities: walking, A1; standing, A2; sitting, A3; jogging, A4; running, A5; walking upstairs, A6; walking downstairs, A7; still, A8; biking, A9; driving a car, A10; in vehicle, A11; jumping, A12; using elevator up, A13; using elevator down, A14; vacuuming, A15; laying, A16; phone on table/detached, A17; washing dishes, WD; ironing, IR; brushing teeth, BT; hair drying, HD; flushing the toilet, FTT; boarding, BD; unknown.
